# Anti-inflammatory and analgesic potential of minor cannabinoids in vivo

**DOI:** 10.1186/s42238-025-00384-7

**Published:** 2026-02-12

**Authors:** S. O. Vanegas, T. F. Gamage, J. Maturano, D. Sarlah, S. G. Kinsey

**Affiliations:** 1 School of Nursing, University of Connecticut, 231 Glenbrook Rd U-4026, Storrs, CT 06269 USA; 2 Department of Psychological Sciences, University of Connecticut, 231 Glenbrook Rd U-4026, Storrs, CT 06269 USA; 3 Department of Neuroscience and Physiology, State University of New York Upstate Medical University, Syracuse, NY USA; 4 Department of Chemistry, Rice University, Houston, TX USA

**Keywords:** Minor cannabinoid, Cannabinol, Cannabichromene, Cannabicyclol, Cannabigerol, Pain, Inflammation

## Abstract

**Background:**

The cannabis plant produces many bioactive compounds, including the major cannabinoids THC and CBD, and many lesser studied “minor” phytocannabinoids including cannabinol (CBN), cannabichromene (CBC), cannabicyclol (CBL), and cannabigerol (CBG). These compounds are touted for various ailments, including pain, inflammation, and anxiety, but experimental data on their effects are lacking, especially that of CBL, which has yet to be assessed in vivo.

**Methods:**

To assess in vivo activity, adult male and female C57BL/6J mice were administered each compound and tested repeatedly in the tetrad battery. The potential analgesic effects in chronic pain states were assessed using the lipopolysaccharide (LPS)-induced hindpaw inflammatory pain and chronic constriction injury (CCI) neuropathic pain paradigms. Lastly, to address common psychological comorbidities of pain, CBN, CBL, and CBG were assessed in the tail suspension and marble burying tests.

**Results:**

Cannabinol (≥ 25 mg/kg) induced classic cannabinoid effects, including acute antinociception. These effects were differentially and partially blocked by selective antagonism of CB_1_, adenosine A_2A_, or TRPV1 receptors. CBL (≥ 50 mg/kg) induced hypothermia that was fully blocked by A_2A_ antagonism but had no apparent CB_1_-mediated activity. LPS-induced edema and paw proinflammatory cytokine levels were reduced by either CBN or CBL (100 mg/kg). CCI-induced cold allodynia was attenuated by either CBN (≥ 50 mg/kg) or CBL (100 mg/kg), but only at high doses that also induce catalepsy and hypothermia. None of these minor cannabinoids displayed anxiolytic- or antidepressant-like activity without concomitant locomotor effects.

**Conclusions:**

Together, these findings suggest that CBN produces anti-inflammatory effects via cannabinoid receptor-dependent and -independent pathways, whereas CBL acts primarily through CB receptor-independent mechanisms.

**Supplementary Information:**

The online version contains supplementary material available at 10.1186/s42238-025-00384-7.

## Introduction

The endocannabinoid system regulates many biological processes, including metabolism, sleep, stress, immune function, and neuroendocrine activity ​(Finn 2010; Lu and Mackie 2021; Svíženská et al. 2008) warranting its attraction as a research target for novel pharmacotherapies. Cannabis plants contain a complex combination of over 550 identified chemical compounds (Ahmed et al. 2015; Rock and Parker 2021), some of which act on the cannabinoid receptors (i.e., CB_1_ and CB_2_). Specifically, 125 cannabinoid compounds have been isolated from the cannabis plant (ElSohly and Gul 2014; Radwan et al. 2021). Delta-9-tetrahydrocannabinol (Δ^9^-THC) and cannabidiol (CBD) are the most abundant and well-studied of these cannabis phytoconstituents and are thus referred to as the ‘major cannabinoids.’

Beyond Δ^9^-THC and CBD, some of the most prevalent cannabinoids found in cannabis flowers are delta-8-tetrahydrocannabinol (Δ^8^-THC), cannabigerol (CBG), cannabinol (CBN), and cannabichromene (CBC) (Al Bakain et al. 2020; ElSohly and Gul 2014; Mechoulam 2005). Additionally, cannabicyclol (CBL), cannabinodiol (CBND), cannabielsoin (CBE), and cannabitriol (CBT) may be present in negligible concentrations (Morales et al. 2017). The marketing and popularity of minor cannabinoid products, especially in areas where Δ^9^-THC use is restricted, far outstrips empirical evidence to support the purported therapeutic effects, or potential physiological mechanisms of these minor cannabinoids.

CBN and CBC, which are natural breakdown products of Δ^9^-THC and cannabichromenic acid, respectively, have relatively higher in vitro receptor affinity for CB_2_ over CB_1_ (De Petrocellis et al. 2011; Morales et al. 2017; Rhee et al. 1997; Rosenthaler et al. 2014). However, the in vivo effects of CBN are inconsistent in the tetrad battery, a classic whole animal screen for CB_1_ agonist activity that includes tests of catalepsy, antinociception, body temperature, and locomotor activity. For example, CBN reduced locomotor activity but did not induce other tetrad effects in male Swiss mice (El-Alfy et al. 2010), whereas all tetrad behaviors except for catalepsy were observed in male and female ICR mice (Schwarz et al. 2024). These variable results obscure whether CBN induces cannabimimetic effects in vivo, or whether they may occur via CB_1_. In addition to acting on the cannabinoid receptors, CBN and CBC act on temperature-sensitive TRP channels, including TRPV1, consistent with analgesic potential (Cascio and Pertwee 2014; De Petrocellis et al. 2011). Indeed, CBN attenuates mechanical allodynia in a mouse chemotherapy-induced peripheral neuropathy model (Schwarz et al. 2024). Similarly, CBC induces antinociceptive effects in the mouse tail-flick assay (Davis and Hatoum 1983) attributed to activity at CB_1_, adenosine A1, and TRPA1, but not TRPV1 receptors (Maione et al. 2011). In contrast, beyond the structure and biosynthetic pathway of CBL, a degrative product of CBC (Crombie et al. 1968; Ferraro and Umstead 2023; Kane et al. 1984), very little is known about this compound. CBL appears to have antiviral effects against SARS-CoV-2 spike protein fusion in Vero cells (Classen et al. 2024), and acts as a potent positive allosteric modulator of 5-HT_1A_ in vitro (Haghdoost et al. 2025). Yet, there are no reports of its pharmacological effects in vivo.

The overall goal of the present study was to determine the cannabimimetic, analgesic, anxiolytic and antidepressive effects of the minor cannabinoids CBN, CBC, and CBL. To this end, each compound was screened for cannabimimetic effects using the tetrad battery, and these effects were challenged via pharmacological blockade of CB_1_, adenosine A_2A_, and TRPV1 receptors. The analgesic and anti-inflammatory potential of each compound was assessed in the lipopolysaccharide (LPS) inflammatory pain and chronic constriction injury (CCI) neuropathic pain models. Chronic pain is comorbid with psychiatric conditions (Roughan et al. 2021). Identifying drugs that reduce both physical and emotional aspects of pain would improve treatment outcomes and reduce reliance on polydrug therapy, thereby lowering the risk for side effects and harmful drug interactions. Thus, each compound was assessed using common anxiolytic and antidepressant drug screens.

## Materials and methods

### Animals

Adult (> 8-weeks-old) male and female C57BL/6J mice (The Jackson Laboratory; Bar Harbor, ME) were group-housed (4–5 per cage) in polysulfone plastic cages (Optimice: C79100PFF) on aspen chip bedding with food and water available *ad libitum*. All mice were drug- and experimentally-naïve unless explicitly stated otherwise. Mice were housed in a temperature (21 ± 1 °C) and humidity (40 ± 5%) controlled room, on a 12:12 h light: dark cycle (lights on at 0700) in an AAALAC accredited facility. Mice were stratified by sex before random assignment, within each cage, to treatment group, using a random number generator, such that each cage contained mice from at least two different treatment groups (i.e., no cage contains only vehicle- or drug-treated mice). A power analysis of preliminary data, using a power of 0.8, an alpha of 0.05, and medium effect sizes, indicated a required total sample size of 8 mice. All experiments were carried out during the light phase, starting at approximately 0800 by trained technicians who were blinded to treatment conditions. For the locomotor and marble burying assays, test apparatuses were placed inside sound-attenuating test chambers outfitted with a fan and white LED/infrared lighting. The Institutional Animal Care and Use Committee at the University of Connecticut approved all experimental protocols.

### Drugs

The minor phytocannabinoids cannabinol (CBN), cannabichromene (CBC), cannabicyclol (CBL), and cannabigerol (CBG) were synthesized by the Sarlah lab, as previously described (Dennis et al. 2022; Yeom et al. 2013) and purified by column chromatography to purity > 95% (HPLC and NMR). CBN and CBL were tested in all experimental paradigms, CBC was tested in all paradigms except the lipopolysaccharide (LPS) and anxiety-/depressive-like behavior experiments, and CBG was only tested in the anxiety-/depressive-like behavior experiments. The selective CB_1_ receptor antagonist SR141716A (rimonabant), the synthetic CB_2_-selective antagonist SR144528 (“SR2”), the adenosine A_2A_ receptor antagonist istradefylline, the TRPV1-selective antagonist capsazepine, and the synthetic glucocorticoid dexamethasone were purchased from Cayman Chemical (Ann Arbor, MI). All drugs were dissolved in a vehicle solution of 5% ethanol, 5% Kolliphor EL (Sigma-Aldrich), and 90% normal saline (Kinsey and Cole 2013). All solutions were injected at a volume of 10 µL/g body mass.

### Dose selection

**Fig. 1:**
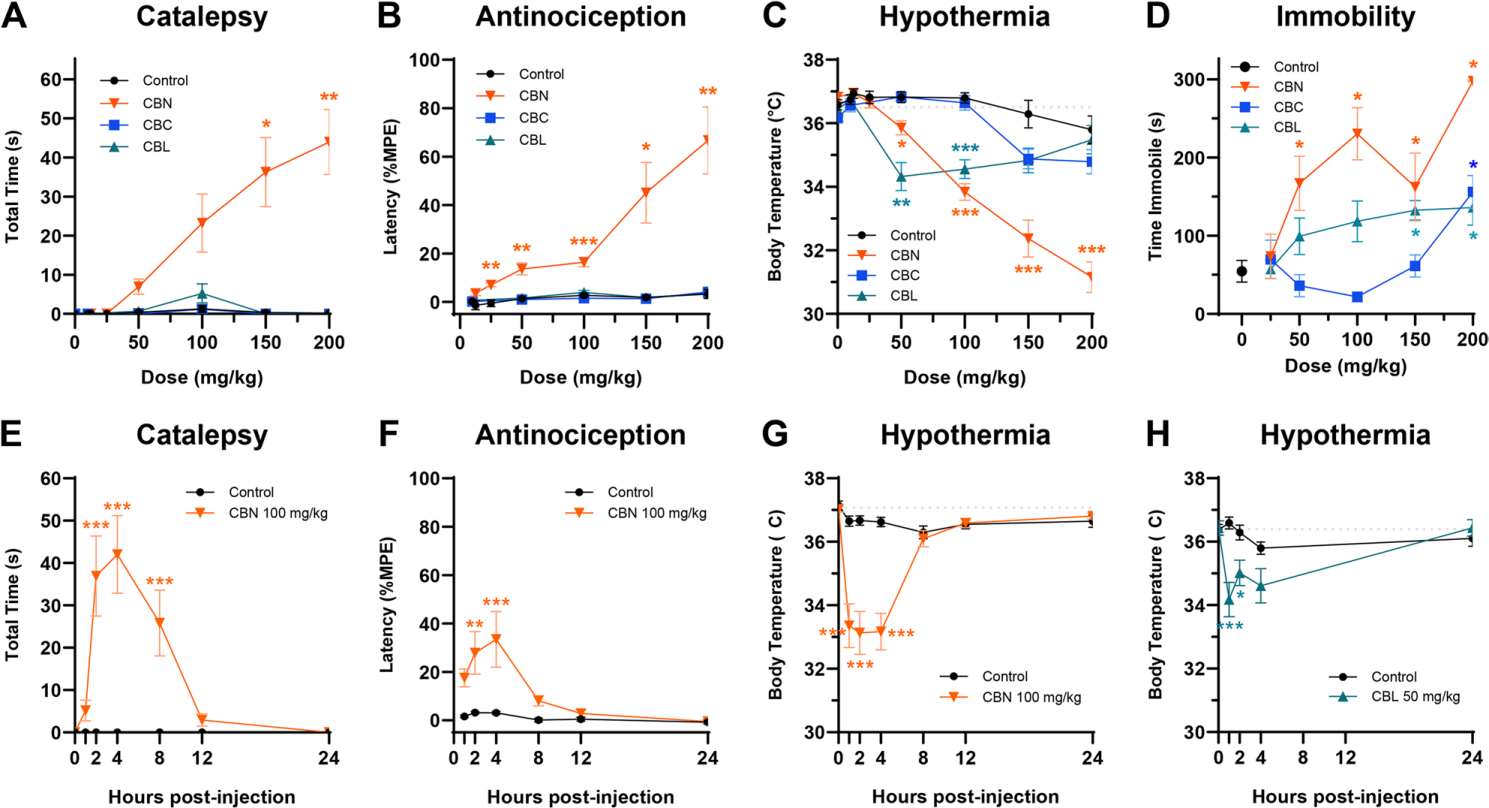
Dose-response and time course effects of minor cannabinoids in the tetrad. Only CBN induced (**A**) catalepsy and (**B**) antinociception. CBN and CBL induced (**C**) hypothermia, whereas (**D**) all three compounds induced dose-dependent immobility. **E**-**G** The effects of CBN peaked at 4 h and persisted at least 4–8 h. **H** CBL-induced hypothermia persisted at least 2 h. Gray dotted line indicates average baseline body temperature. Data are expressed as mean ± SEM (*n* = 9–10 [4–5 M/4–5 F]). *-***p < 0.05-0.0005 vs. vehicle control

Based on previous literature, we used the highest dose of each antagonist that blocks the action of an agonist without inducing effects per se: rimonabant 3 mg/kg (Schlosburg et al. 2011), istradefylline 3.2 mg/kg (LaVigne et al. 2021), capsazepine 5 mg/kg (Nguyen et al. 2010). In the dose response experiment (Fig. 1), we observed that 200 mg/kg of the minor cannabinoids was not well tolerated, thus doses of ≤ 150 mg/kg were used in subsequent testing.

### Tetrad battery

The “tetrad” is a well-characterized battery of four assays used to evaluate the effects of cannabinoid agonists (Lichtman et al. 2001). It consists of assessments of catalepsy, antinociception, core body temperature, and spontaneous locomotor activity. Mice were acclimated to the test room for a minimum of 1 h before testing (Grim et al. 2016; Schlosburg et al. 2009)​.

- *Catalepsy* was assessed first by gently laying the forepaws of each mouse over a metal bar elevated 3 cm above the benchtop. Total latency to move a forepaw off the bar was recorded over three trials, with a maximum cutoff of 60 s (Long et al. 2009).
- *Antinociception* was measured immediately following the catalepsy test by gently placing each mouse headfirst into a small, padded bag and immersing the 1 cm distal tip of the tail into a 56 °C water bath (Falenski et al., 2010). Latency to withdraw the tail from the water was recorded, with a maximum cutoff of 10 s. Tail immersion data is represented as a percent of the maximum possible effect (%MPE; [(measured response - baseline response)/(10 s - baseline response)x100]) (Kaski et al. 2019).
- *Hypothermia* was assessed immediately following the tail immersion test by taking rectal temperature using a microprobe thermocouple thermometer designed for use with mice (BAT-12 with RET-3 probe, Physitemp Instruments Inc., Clifton, NJ, USA).
- *Spontaneous locomotor activity* was measured last, by placing individual mice into a transparent plastic test chamber (18 cm W x 28 cm L x 12 cm H) inside a sound-attenuating chamber outfitted with a fan, white LED/infrared lighting, and an overhead video camera. Testing lasted 5 min and locomotor activity was scored in real-time using ANY-maze video tracking software (Stoelting, Wool Dale, IL, USA). Time immobile was determined by setting the tracking parameters to a latency of 1200 ms for 90% of the mouse image pixels (Trexler et al. 2020). In some cases where mice were repeatedly tested (i.e., time course experiment), locomotor activity was not measured, because mice quickly habituate to locomotor testing.

### Acute drug effects

To evaluate the dose-dependent acute effects of each compound, 4–5 male and 4–5 female mice (*n* = 9–10 for each compound) were baselined for catalepsy, tail withdrawal latency in the tail immersion test, and body temperature and then injected (i.p.) with cumulative doses of CBN (12.5, 12.5, 25, 50, 50, & 50 mg/kg), CBC (10, 40, 50, 50, & 50 mg/kg), or CBL (10, 40, 50, 50, & 50 mg/kg) to create a dose-response curve (CBN final doses: 12.5, 25, 50, 100, 150, & 200 mg/kg; CBC/CBL final doses: 10, 50, 100, 150, & 200 mg/kg) 1 h prior to subsequent tetrad testing (i.e., 60 min interval between each dose). Control mice received repeated injections of vehicle. The tetrad dose-response experiment was the only instance where cumulative dosing was used. Locomotor activity was measured in separate groups of naïve mice (i.e., each mouse only tested once; *n* = 8 for each dose; 128 total) to avoid habituation.

To determine the time course of cannabinol-induced cannabimimetic effects, a separate group of naïve mice (*n* = 10[5 M/5F] per group) were baseline.

tested for catalepsy, tail withdrawal latency in the tail immersion test, and body temperature and then injected (i.p.) with a single dose of CBN (100 mg/kg) or vehicle and tested repeatedly (1, 2, 4, 8, 12, 24 h post-injection) in a modified tetrad battery.

(i.e., bar test, tail immersion test, and body temperature). This procedure was also used to determine the time course of CBL-induced hypothermia (50 mg/kg, i.p.) wherein only body temperature was repeatedly tested at 0, 1, 2, and 4 h post-injection (*n* = 10[5 M/5F] per group).

### Receptor mechanism challenge

To probe receptor mechanisms by which CBN, CBC, and CBL induce tetrad effects, a separate group of naïve mice (*n* = 9–10 [4–5 M/4–5 F]) was administered the CB_1_-selective antagonist rimonabant (3 mg/kg, i.p.), the A_2A_ receptor-selective antagonist istradefylline (3.2 mg/kg) (LaVigne et al. 2021), the TRPV1 antagonist capsazepine (5 mg/kg) (Nguyen et al. 2010), or vehicle 30 min prior to administration (i.p.) of CBN, CBC, CBL (100–150 mg/kg) or vehicle. For the rimonabant and istradefylline challenges, testing was conducted 1 h after administration of the minor cannabinoids or vehicle. A 2 h pretreatment was used for the capsazepine challenge to circumvent the hyperreflexive effects of CBN observed in previous experiments.

### Lipopolysaccharide (LPS)-induced paw edema

Inflammatory pain was induced by injecting 25 µg LPS from Escherichia coli 026:B6 (Sigma, St. Louis, MO, USA) in 50 µl of sterile normal saline into the plantar surface of each hind paw (DeLong et al. 2010; Kanaan et al. 1996). Control mice received 50 µl of saline into each hind paw. Paw thickness was measured both before and 24 h after LPS injection, using digital calipers (Mitutoyo, Japan) and expressed to the nearest 0.01 mm. Additionally, thermal preference in a thermal gradient ring were measured 24–26 h post-LPS injection. The synthetic glucocorticoid dexamethasone was used as a positive control. Experimental compounds (i.e., CBN, CBL, or dexamethasone) were injected 1 h before and 6 and 23 h after LPS administration (i.e., 3 injections total). CB_1_/CB_2_ antagonists (rimonabant or SR144528) were injected 30 min before each CBN injection.

### Thermal Gradient Ring (TGR) test

An automated Thermal Gradient Ring (Ugo Basile, Italy) is an enclosed, conductive ring connecting two hot/cold plates that create a temperature differential across the ring (i.e., 20–40 °C) (Rodriguez et al. 2024; Vanegas et al. 2024). Individual mice were placed in the 23 ± 1 °C zone (i.e., room temperature) on alternating sides of the ring and allowed to freely explore for 16 min. Mouse location and movement were tracked using an overhead camera and analyzed in real-time using ANY-maze software. Dependent variables included the weighted average of temperature/zone occupancy (i.e., “thermal preference”) and distance traveled.

### Paw cytokine quantification (Ella)

At the conclusion of all behavioral testing, hind paw soft tissue was collected, snap-frozen in liquid nitrogen, and stored at −80 °C until assay. Samples were homogenized using a Tissue Tearor (Bartlesville, OK) in 1 mL ice-cold phosphate-buffered saline and centrifuged at 4 °C and 7000 g for 15 min. The supernatant was collected and used for quantification of cytokines (IL-1β, IL-6, & TNF-α) in triplicate via Ella multiplex ELISA cartridges (ProteinSimple, Minneapolis, MN, USA). Cytokine levels were standardized to total wet weight (g) of each sample.

### Chronic Constriction Injury (CCI) of the sciatic nerve

CCI surgery was performed as described previously (Crowe et al. 2015; Crowe and Kinsey 2017; Grim et al. 2014)​. Briefly, under isoflurane anesthesia, an incision was made in the skin posterior to the femur, and the sciatic nerve was loosely ligated using a single sterile, 5 − 0 braided silk suture. The surrounding muscle and skin were sutured with sterile 6 − 0 nylon monofilament suture (Ethilon) with a P-1 11 mm 3/8c reverse cutting needle. Allodynia in the paw ipsilateral to CCI was evident 7–10 days post-surgery. Thus, nociceptive testing began no earlier than 10 days after surgery, as previously described (Crowe et al. 2015; Crowe and Kinsey 2017)​. To reduce animal numbers, a total of 40 (20 M/20F) mice were used in the CCI experiments. Mice received a maximum of three treatments, and a washout period of at least one week was given between treatments.

### Mechanical and cold allodynia

Individual mice were placed on an aluminum wire mesh table and acclimated for at least 1 h before measuring cold allodynia in the acetone test. A small, fixed amount (10 µl) of acetone was pipetted onto the plantar surface of each hind paw, after which the total time spent lifting the paw off the mesh surface was recorded using a stopwatch for up to 30s. The average across three trials was used for analyses. Immediately following the acetone test, mechanical allodynia was assessed. The hind paws of each mouse were stimulated with calibrated von Frey filaments using a modified version of the simplified up-down (SUDO) method (Bonin et al. 2014). The plantar surface of each hind paw was stimulated 3–5 times with each filament (0.07–6.0 g), starting with the 1 g filament and increasing or decreasing by two sequential filaments depending on whether the mouse responded by clutching and/or lifting the paw. Paw lifting in response to 3 or more stimulations was coded as a positive response. When there was a change in the response (i.e., paw lift in response to a higher filament or no response to a lighter filament), the next lightest or heaviest filament, respectively, was used to detect the sensory threshold. The paw withdrawal threshold was defined as the thickest filament used that did not elicit a positive response.

### Minor cannabinoid effects on anxiety- and depressive-like behaviors

Pain is multifaceted and stress-inducing. Individuals with chronic pain conditions are more likely to develop mental health disorders, including anxiety and depression, which may exacerbate pain perception (Roughan et al. 2021; Vinall et al. 2016). In consideration of this significant comorbidity, the effects of minor cannabinoids were evaluated in the below anxiety- and depressive-like behavioral models. Of note, the effects of CBC (El-Alfy et al. 2010), but not cannabigerol (CBG), have previously been studied in the tail suspension test. CBG increases rat open arm time in the elevated plus maze (Mendiguren et al. 2023). Thus, we determined the effects of CBN, CBL, and CBG in the following screens for anxiolytic and antidepressant drugs.

### Tail suspension test

The tail suspension test was performed as previously described (Trexler et al. 2018). Mice were suspended by their tails using adhesive tape attached to a horizontal bar placed approximately 40 cm above the benchtop and video recorded for 6 min. The amount of time the mice actively struggled was hand-scored by a trained and blinded observer. A subset of 6 videos was scored by a second observer to ensure inter-rater reliability (r^2^ = 0.98). Active struggling was defined as one or more legs repeatedly kicking within one second or arching of the spine. Swinging motions or head movement per se was not scored as struggling. It is important to note that increased immobility while briefly suspended by the tail is associated with higher levels of depressive-like activity, which can be reversed using antidepressants (Steru et al. 1985). Given that mice quickly habituate to this test, separate groups of experimentally naïve mice were used to test each dose (*n* = 8–10 per dose).

### Marble burying test

The marble burying test was performed as a standalone measure as previously described (Broekkamp et al. 1986; Kinsey et al. 2011b; Trexler et al. 2018). Clear, plastic cages (18 cm W x 28 cm L x 12 cm H) filled with 5 cm of Teklad Aspen Sani-Chip bedding (7090 A; Envigo, Indianapolis, IN, USA) and a 4 × 5 array of 20 clear glass marbles was laid gently across the top of the leveled bedding in a grid-like fashion. 60 min following injection of experimental compounds, each mouse was individually placed facing into the front, right-hand corner of the cage and allowed to explore freely for 20 min. At the end of the test, each mouse was quickly and carefully removed, and the number of unburied marbles (≥ 1/3 of the surface showing) was recorded and subtracted from the 20 total marbles. activity was simultaneously recorded for the duration of the test by a camera mounted on the top of the test chamber and analyzed in real time using ANY-maze (Stoelting, Wood Dale, IL, USA). Dependent variables included time immobile and the number of marbles buried. Time immobile was determined by setting the tracking parameters to a latency of 1200 ms for 90% of the mouse image pixels (Trexler et al. 2020).

### Data and statistical analyses

Time course and dose-response main effects of each target compound were compared using two-way mixed ANOVA, with drug treatment as a between-subjects variable and time or drug dose as a within-subject variable. Data from the receptor antagonist challenge and LPS experiments were analyzed using one-way ANOVA, followed by Bonferroni *post hoc* comparisons, with drug treatment as the between-subjects independent variable. Because this study was not designed to analyze sex effects, it was underpowered to detect true sex differences, and thus data are presented collapsed across sexes which may increase variability and mask some effects. All data are represented as mean ± SEM and results were considered significant if *p* < 0.05.

## Results

### Cannabimimetic effects of CBN persist for at least 4 h

To assess acute cannabimimetic effects, mice were administered CBN (12.5–200 mg/kg, i.p.), CBC (10–200 mg/kg, i.p.), or CBL (10–200 mg/kg, i.p.) 60 min prior to testing in the tetrad battery. Only CBN produced all four tetrad effects (Table S1). That is, CBN induced catalepsy at ≥ 150 mg/kg (Fig. 1A), antinociception at ≥ 50 mg/kg (Fig. 1B), hypothermia at ≥ 50 mg/kg (Fig. 1C), and immobility at 100 and 200 mg/kg (Fig. 1D). CBC induced immobility at the highest dose tested (200 mg/kg) (Fig. 1D) but had no effect on catalepsy, antinociception, or hypothermia. CBL induced hypothermia at 50–100 mg/kg (Fig. 1C), and immobility at ≥ 150 mg/kg (Fig. 1D), but had no effect on catalepsy or antinociception. Baseline measurements (mean ± SEM) for each test were: bar test (0 ± 0 s), tail immersion test (0.99 ± 0.09 s), body temperature (36.5 ± 0.56 °C), and time immobile (54.65 ± 41.44 s). CBN-induced (100 mg/kg, i.p.) catalepsy (Fig. 1E), antinociception (Fig. 1F), and hypothermia (Fig. 1G) peaked at 4 h and persisted for at least 4–8 h post-injection, whereas CBL-induced hypothermia (50 mg/kg, i.p.) peaked at 1 h and persisted at least 2 h post-injection (Table S2). The time course effects of immobility were not assessed for any of the cannabinoids, given that mice could not be repeatedly tested in the locomotor test.

### CBN and CBL act via CB_1_-dependent and -independent mechanisms

**Fig. 2:**
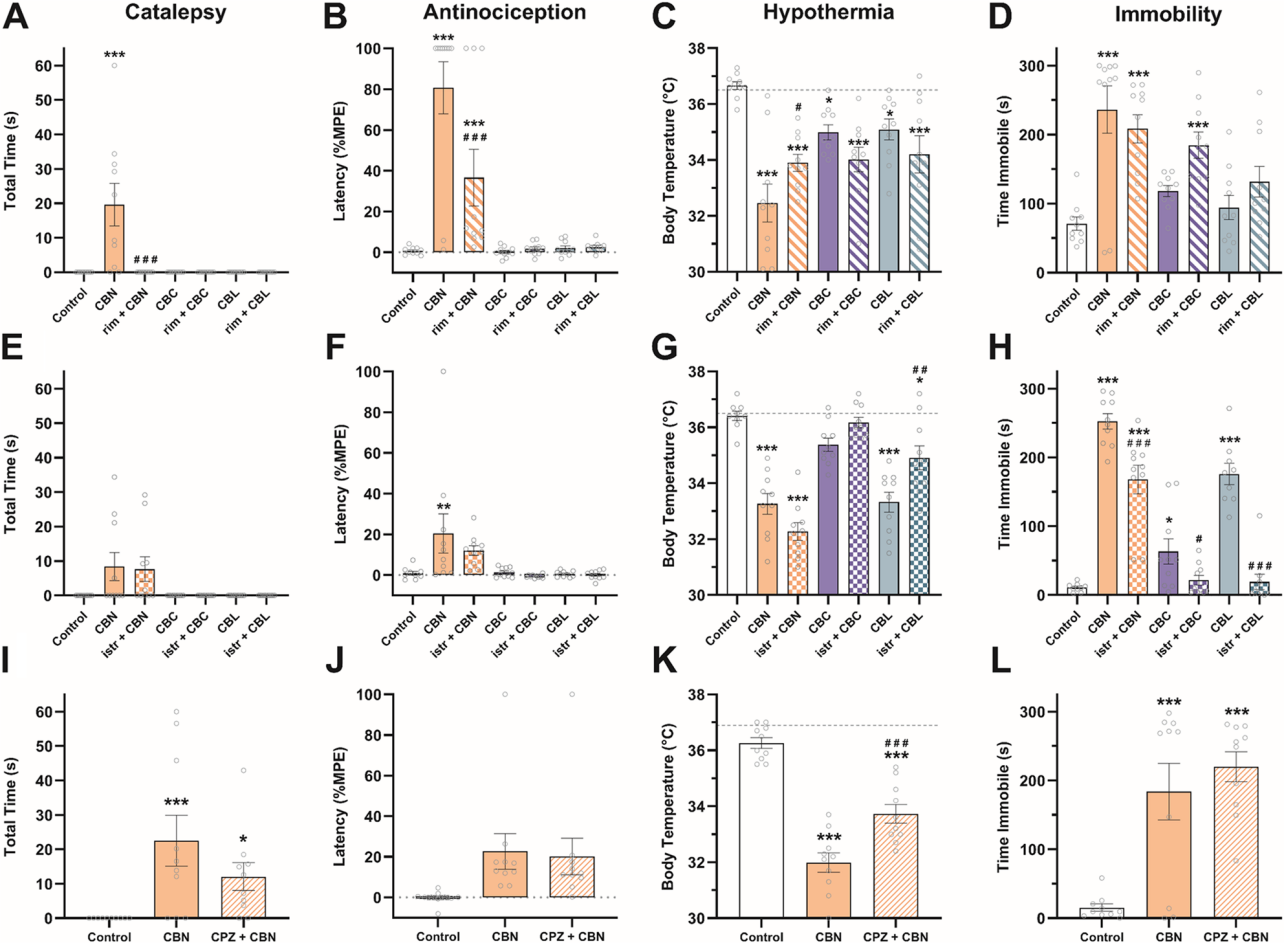
CBN and CBL effects occur via multiple receptor mechanisms. The tetrad effects of CBN, CBC, and CBL were challenged using (**A**-**D**) the CB_1_**-**selective antagonist rimonabant (3 mg/kg, i.p.) and (**E**-**H**) the adenosine A_2A_-selective antagonist istradefylline (3.2 mg/kg, i.p.). **I**-**L** CBN’s effects were also challenged using the TRPV1-selective antagonist capsazepine (5 mg/kg, i.p.). Dashed line in C, G, & K indicates average baseline body temperature. Data are expressed as mean ± SEM (*n* = 9–10 [4–5 M/4–5 F]). *-***p < 0.05-0.0005 vs. vehicle control, #-###*p* < 0.05-0.0005 vs. drug-matched control

To further probe potential CB_1_ receptor activity, a separate group of mice was pretreated (i.p.) with the CB_1_ antagonist rimonabant (3 mg/kg) (Kinsey et al. 2009; Schlosburg et al. 2011) 30 min before CBN (150 mg/kg), CBC (150 mg/kg), or CBL (100 mg/kg) and tested in the tetrad battery. One-way ANOVA revealed a main effect of treatment for each test: catalepsy (Fig. 2A), antinociception (Fig. 2B), hypothermia (Fig. 2C), and immobility (Fig. 2D). Rimonabant pretreatment blocked CBN-induced catalepsy (*p* < 0.0001, Table S3) and partially blocked the antinociceptive (*p* = 0.0008 vs. control, *p* < 0.0001 vs. CBN) and hypothermic effects of CBN (*p* < 0.0001 vs. control, *p* = 0.0267 vs. CBN) but had no effect on immobility (*p* = 0.34) indicating that the tetrad effects of CBN are not completely CB_1_-mediated. Rimonabant had no effect on CBL-induced hypothermia (*p* = 0.17) but potentiated CBC-induced immobility (*p* = 0.0245). CBC also induced hypothermia in this experiment, which was not affected by rimonabant pretreatment (*p* = 0.13).

Because the adenosine A_2A_ receptor mediates some tetrad effects of terpenoids found in cannabis (LaVigne et al. 2021), a separate group of mice was pretreated (i.p.) with the A_2A_ antagonist istradefylline (3.2 mg/kg) (LaVigne et al. 2021) 30 min before CBN (100 mg/kg), CBC (100 mg/kg), or CBL (100 mg/kg). Istradefylline had no effect on catalepsy (*p* = 0.79; Fig. 2E) or antinociception (*p* = 0.13; Fig. 2F). However, istradefylline increased CBN-induced hypothermia (*p* = 0.0264; Fig. 2G) and partially blocked CBN-induced immobility (*p* < 0.0001; Fig. 2H). The hypothermic effects of CBL (*p* = 0.0005; Fig. 2G) and locomotor effects of CBC and CBL (*p* = 0.0344 & *p* < 0.0001; Fig. 2H) were also blocked by istradefylline.

The hypothermic effects of CBN were challenged by pretreatment with the TRPV1 antagonist capsazepine (5 mg/kg, i.p.) (Kinsey et al. 2009; Nguyen et al. 2010) 30 min prior to CBN (100 mg/kg) and tested in the tetrad battery. The main effect of drug treatment for each test are as follows: catalepsy (Fig. 2I), tail immersion (Fig. 2J), core body temperature (Fig. 2K), and time immobile (Fig. 2L). Capsazepine partially blocked CBN-induced hypothermia (*p* < 0.0001 vs. control, *p* = 0.0003 vs. CBN) but had no effect on CBN-induced catalepsy (*p* = 0.14) or immobility (*p* = 0.35).

### CBN blocks LPS-induced paw inflammation and cytokine level increase via cannabinoid receptor-independent mechanisms

**Fig. 3:**
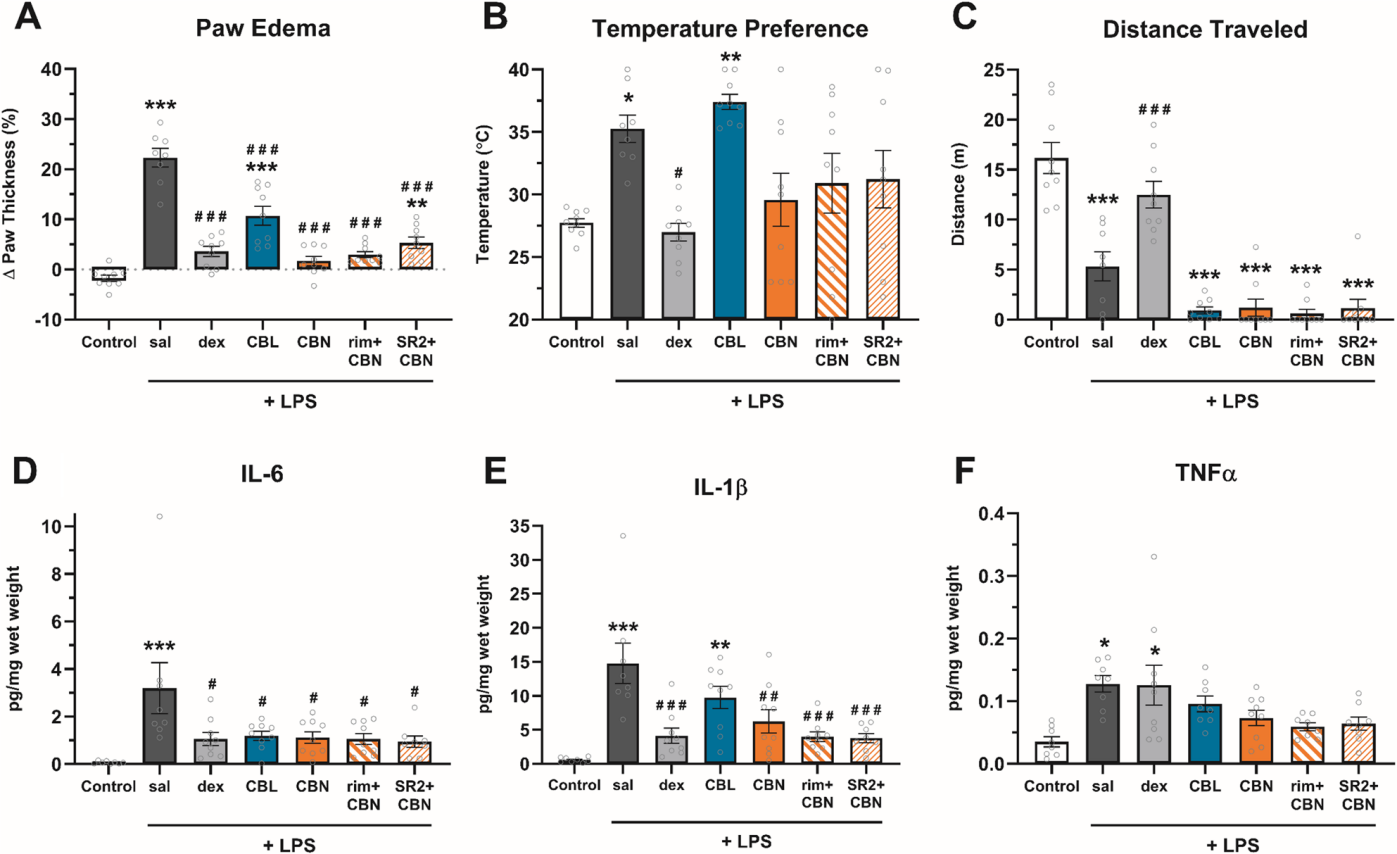
CBN and CBL block LPS-induced edema and inflammation. The effects of CBL and CBN (100 mg/kg, i.p.) on LPS-induced (**A**) paw edema, (**B**) preference for warmth, (**C**) locomotor effects in the TGR, and paw tissue levels of (**D**) IL-6, (**E)** IL-1β, and (**F**) TNF-α. Data are expressed as mean ± SEM (*n* = 8–9 [4–5 M/3–5 F]). *-***p < 0.05-0.0005 vs. vehicle control, #-###*p* < 0.05-0.0005 vs. saline + LPS

To assess anti-inflammatory potential, CBN (100 mg/kg), CBL (100 mg/kg), dexamethasone (2 mg/kg), or vehicle was administered (i.p.) 1 h before, and 6 and 23 h after, injecting lipopolysaccharide (LPS) into both hind paws (Table S4). At 24 h-post LPS, there was a main effect of treatment on paw thickness (Fig. 3A), thermal preference (Fig. 3B), and distance traveled in the thermal gradient ring (Fig. 3C). CBN prevented and CBL reduced LPS-induced paw edema (*p* < 0.0001). CBN-treated mice preferred the same temperatures as controls but also traveled less in the thermal gradient ring (TGR) (*p* < 0.0001), which may have increased variance in thermal preference. CBL also reduced distance traveled in the TGR (*p* < 0.0001) but did not block LPS-induced warmth preference (*p* > 0.99).

Either CBN (*p* = 0.0272) or CBL (*p* = 0.0403) blocked LPS-increased paw levels of IL-6 (Fig. 3D), but only CBN (*p* = 0.0048) blocked LPS-increased levels of IL-1β (Fig. 3E). There was a main effect of treatment for levels of TNF-α (Fig. 3F), and Bonferroni post hoc analyses revealed that neither CBN (*p* > 0.99) nor CBL (*p* = 0.29) differed significantly from controls.

To probe whether the anti-inflammatory effects of CBN are cannabinoid receptor-dependent, two separate groups of CBN-treated mice were pretreated (i.p.) with either rimonabant (3 mg/kg) or SR2 (3 mg/kg) 30 min prior to each CBN injection. SR2-treated mice differed from controls (*p* = 0.0027), indicating a partial block of CBN-attenuated paw edema (Fig. 3A). Neither rimonabant nor SR2 influenced any CBN effects in other measures.

### CBN and CBL reverse CCI-induced cold allodynia at doses that induce hyperreflexia and hypothermia

**Fig. 4:**
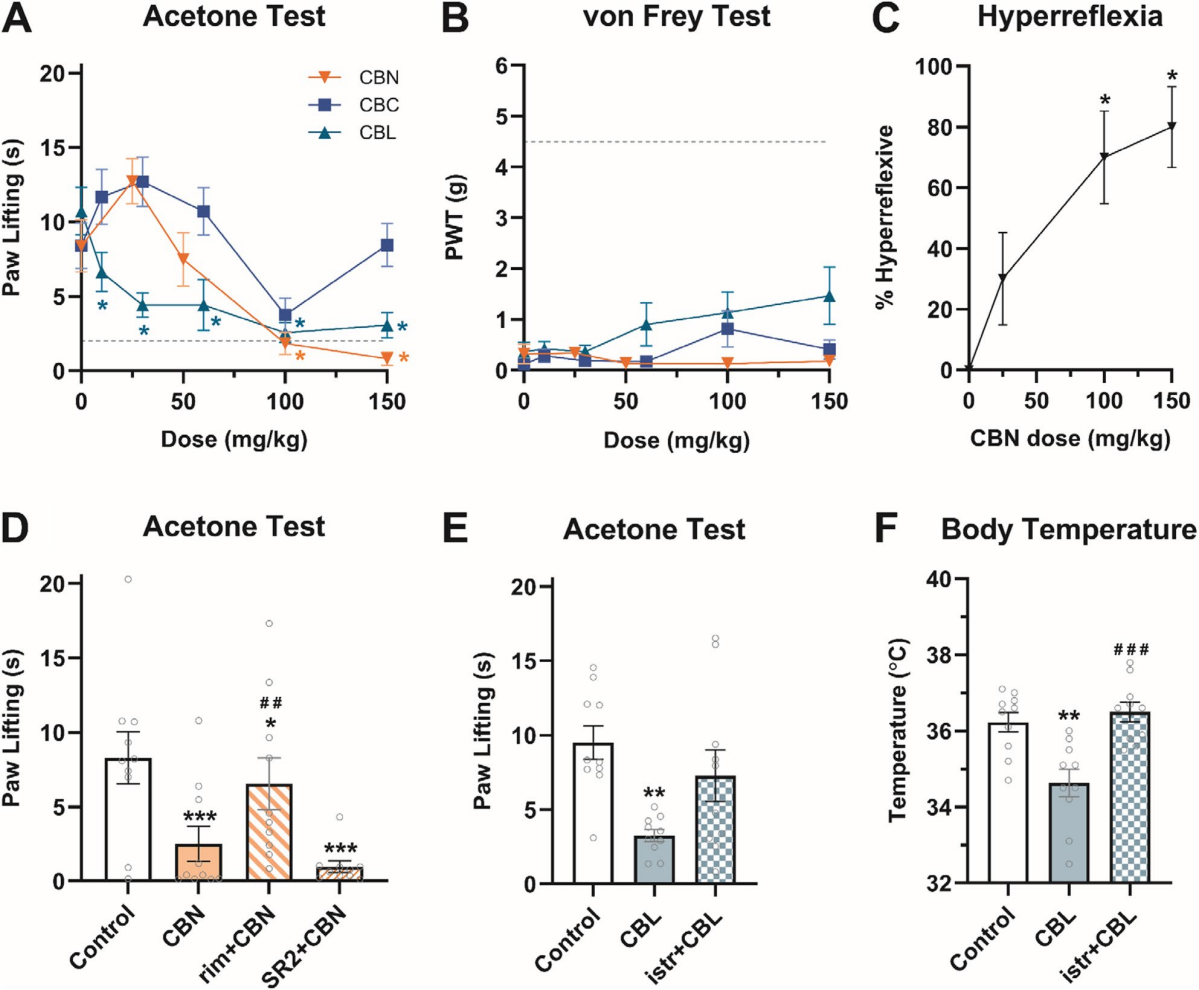
CBN and CBL reduce cold allodynia caused by CCI sciatic nerve injury at hyperreflexive and hypothermic doses. The dose-dependent antiallodynic effects of CBN (25–150 mg/kg), CBC (10–150 mg/kg), and CBL (10–150 mg/kg) were assessed in the (**A**) acetone and (**B**) von Frey tests. **C** Hyperreflexia was also measured in the CBN-treated mice following allodynia testing and is shown here as the percentage of hyperreflexive mice at each dose. **D** The effect of CBN (100 mg/kg) on cold allodynia was challenged by a CB_1_- (rim; 3 mg/kg) and a CB_2_-selective antagonist (SR2; 3 mg/kg). Istradefylline (3.2 mg/kg) was used to challenge the effects of CBL (30 mg/kg) on (**E**) cold allodynia and (**F**) core body temperature. Dashed line in A & B indicates contralateral paw threshold of vehicle controls. Data are expressed as mean ± SEM (*n* = 10 [5 M/5F]). *-***p < 0.05-0.0005 vs. vehicle control, #-###*p* < 0.05-0.0005 vs. drug-matched control (D-F)

Chronic neuropathic pain is particularly resistant to existing analgesics. So, in addition to inflammatory pain, we evaluated the analgesic efficacy of minor cannabinoids in a chronic neuropathic pain model. Mice were subjected to chronic constriction injury (CCI) of the sciatic nerve and CBN (25, 50, 100, 150 mg/kg), CBC (10, 30, 60, 100, 150 mg/kg), or CBL (10, 30, 60, 100, 150 mg/kg) was administered (i.p.) to assess acute antiallodynic potential (Table S5). CBN (≥ 100 mg/kg, i.p.) and CBL (≥ 30 mg/kg, i.p.) reduced CCI-increased paw lifting in the cold allodynia test (Fig. 4A). There was a main effect of CBC treatment on acetone-induced allodynia, although Bonferroni *post hoc* analyses revealed no statistically significant differences of individual doses vs. vehicle. Mechanical allodynia was unaffected by CBN, CBC, or CBL (Fig. 4B**)**. However, it is noteworthy that CBN (≥ 100 mg/kg) also induced hyperreflexia (Fig. 4C), limiting interpretation of the von Frey data.

The antiallodynic effects of CBN (100 mg/kg) in the cold allodynia test were blocked by pretreatment (i.p.) of the CB_1_-selective antagonist rimonabant (3 mg/kg) but not the CB_2_-selective antagonist SR2 (3 mg/kg) (Fig. 4D). In contrast, the A_2A_-selective antagonist istradefylline (3.2 mg/kg, i.p.) blocked the antiallodynic effects CBL (30 mg/kg, i.p.) in the acetone-induced cold allodynia test (Fig. 4E). This dose of CBL caused hypothermia in the tetrad, which could interfere with perception of a cold stimulus. Therefore, hypothermia was assessed in these same mice and was also blocked by istradefylline (Fig. 4F). Given that these effects were apparent at high doses, these data suggest that CBN reduces CCI-induced cold allodynia via a CB_1_ mechanism that overlaps with locomotor deficits, while CBL anti-allodynic effects occur at hypothermic doses that are mediated via A_2A_ receptors.

### No effect of minor cannabinoids on anxiety- or depressive-like behaviors

**Fig. 5:**
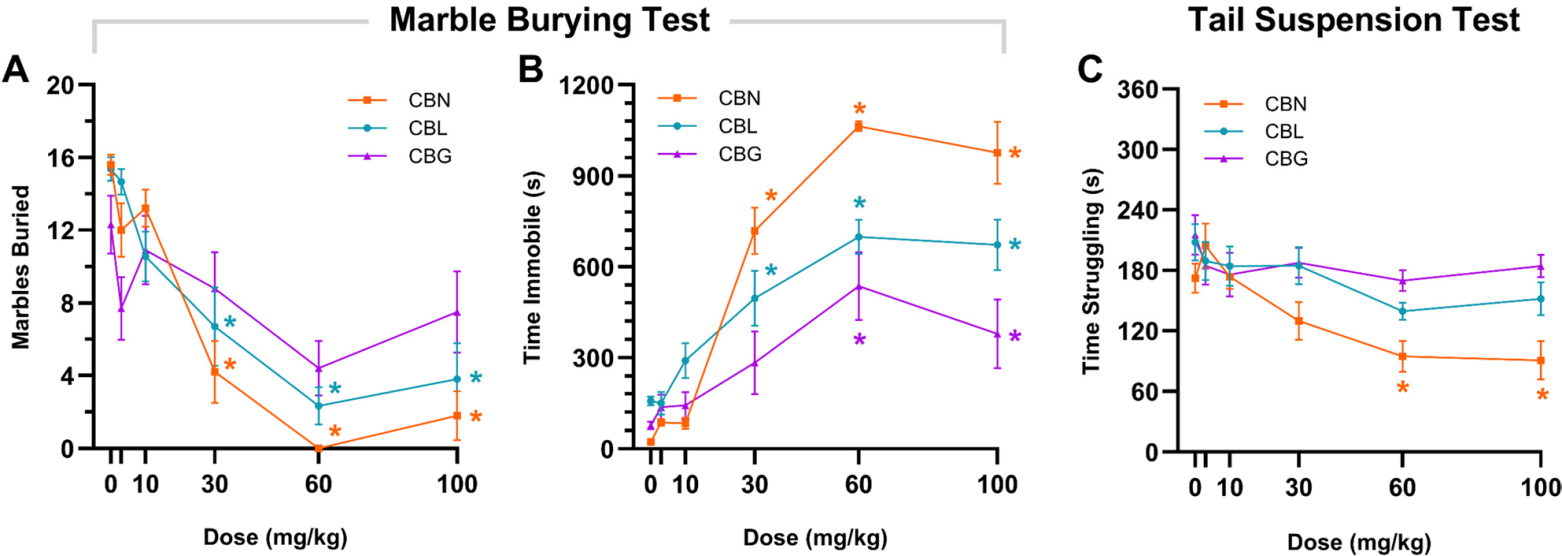
Minor cannabinoids decreased marble burying and struggling in the tail suspension test only at doses that reduce locomotor activity. The dose-dependent effects of CBN, CBL, and CBG (3–100 mg/kg, i.p.) on (**A**) number of marbles buried, (**B**) time immobile in the marble burying test, and (**C**) time struggling in the tail suspension test. Data are expressed as mean ± SEM (n=8–10 [4-5M/4-5F]). *p<0.05 vs vehicle control

Mice were administered (i.p.) a single dose of CBN, CBL, CBG (3, 10, 30, 60, or 100 mg/kg), or vehicle and then assessed 1 h later in the marble-burying behavior and tail suspension tests. CBN and CBL, but not CBG, reduced the number of marbles buried (Fig. 5 A). However, CBN, CBL, and CBG also increased immobility (Fig. 5B), inconsistent with anxiolysis. CBN dose-dependently reduced struggling in the tail suspension test (Fig. 5 C), but neither CBL nor CBG affected time struggling in the tail suspension test.

## Discussion

The goal of the present study was to assess the effects of a subset of minor cannabinoids using established screens for cannabimimetic effects in the tetrad battery, as well as anti-inflammatory and analgesic effects in models of endotoxin-induced inflammatory pain and surgically-induced neuropathic pain. Cannabinol (CBN) was the only minor cannabinoid that induced all four tetrad behaviors, indicative of psychoactive properties consistent with those of CB_1_ agonists, including the major cannabinoid Δ^9^-THC. Relatively high doses of CBN (≥ 50 mg/kg) were required to induce THC-like effects in the tetrad, whereas the tetrad effects of Δ^9^-THC are typically observed at ≥ 10 mg/kg in mice (Vanegas et al. 2022; Wang et al. 2020). Unlike canonical CB_1_ agonists, these acute effects were only partially and differentially blocked by pretreatment with receptor-selective antagonists of CB_1_, A_2A_, or TRPV1. These data align with previous reports that CBN activates both CB_1_ and TRPV1 receptors in vitro (De Petrocellis et al. 2011; Pertwee & Cascio 2014). Thus, it is likely that CBN activates multiple, as yet to be identified receptor systems to produce the antinociception, hypothermia, and immobility observed in the present study. Given the established role of dopaminergic and adrenergic signaling in motor function and their crosstalk with the endocannabinoid system (Li et al. 2023; Mendiguren et al. 2023; Peters et al. 2021; Zikereya et al. 2025), we speculate that D2 or α−2 receptors are favorable targets for further investigation. In support of this idea, CBN inhibits the release of dopamine and norepinephrine in the rat brain, suggesting potential indirect effects on motor activity (Poddar and Dewey 1980).

It is possible that higher doses of rimonabant, istradefylline, and capsazepine were needed to fully block the effects of the high CBN doses used here. Although the antagonist doses selected are well established as sufficient to block the effects of potent agonists, and higher doses would likely introduce confounding effects of the antagonists themselves (LaVigne et al. 2021; Nguyen et al. 2010; Schlosburg et al. 2011). Additionally, while capsazepine is relatively selective and competitive for TRPV1, it has known activity at other receptors and TRP channels. Future work may therefore benefit from using the more selective TRPV1 antagonist SB-366,791 to challenge the effects of CBN (Varga et al. 2005). Another limitation of this work is that the post-injection interval to observe the effects of CBN in the tetrad needed to be extended from 1 to 2 h to minimize hyperreflexive confounds in the catalepsy test. Because hyperreflexia at the 1-hour time point was intermittent in early experiments, the need for this adjustment only became clear after most studies had been completed. As a result, the optimized 2-hour pretreatment interval was implemented only in the final tetrad experiment (i.e., the capsazepine challenge; Fig. 2I–L). Differences in the pretreatment interval of CBN may also contribute to contrasts between the present data and previous reports that did not observe CBN-induced catalepsy (El-Alfy et al. 2010; Schwarz et al. 2024). Specifically, the previous studies measured catalepsy ≤ 30 min post-injection using the slightly different ring test (versus the bar test used here). Our findings indicate that catalepsy is most pronounced between 2 and 4 h post-CBN administration, which may explain why CBN-induced catalepsy has not been observed previously. It is noteworthy that there were some minor differences between the cumulative dosing and the antagonist challenge experiments. For example, CBC induced hypothermia and increased immobility that was statistically significant only in the latter experiment. We speculate this difference may be due to handling stress in the former or from delivering a large bolus of drug in the latter experiment.CBN blocked both lipopolysaccharide (LPS)-increased paw swelling and proinflammatory cytokine levels. SR144528, but not rimonabant, partially blocked the antiedematous effect of CBN, consistent with a partial CB_2_ mechanism. These in vivo data are consistent with CBN having higher affinity for CB_2_ over CB_1_ in vitro (Pertwee & Cascio 2014). Moreover, CB_2_ activation reduces inflammation and release of TNF-α, IL-1β, and IL-6 from immune cells (Capozzi et al. 2022; Kinsey et al. 2011a, b; Nass et al. 2021; Tang et al. 2018). However, blocking either CB receptor had no effect on cytokine levels in hindpaw tissue, suggesting the involvement of additional, CB_2_-independent mechanisms mediating the anti-inflammatory effects of CBN. It is worth noting that a relatively high concentration of LPS was used in the present study, with the goal of inducing not only allodynia but also edema, which may have masked a potential CB_2_ effect. In further support of this idea, the anti-inflammatory effects of other phytocannabinoids have been linked to multiple parallel receptor systems pathways including peroxisome proliferator-activated receptors (PPARs) (Palomares et al. 2020), GPR55 (Balenga et al. 2011; Yang et al. 2016), 5-HT_1A_ (Landucci et al. 2022), several TRP channels (Muller et al. 2018), cyclooxygenase (COX-1/−2) inhibition (Ruhaak et al. 2011; Takeda et al. 2014), and reduced nitric oxide production (Borrelli et al. 2013; Costa et al. 2007), so it is plausible that CBN also acts through one or more of these receptors in addition to cannabinoid receptors. Similarly, CBC inconsistently produced hypothermic effects, and the hypothermic effect of CBL was fully blocked by istradefylline, so we decided that challenging these compounds with capsazepine was not justified.

We observed acute analgesia with CBN in the tetrad battery, so we next evaluated its efficacy in a chronic pain state. CBN reduced chronic constriction injury (CCI)-induced cold allodynia, but only at doses that also caused catalepsy and hypothermia. The effect of CBN on cold allodynia in the CCI model was blocked by rimonabant, but not SR144528, consistent with a CB_1_-mediated mechanism. Surprisingly, CBN had no effect on CCI-induced mechanical allodynia, contrasting previous work that found CBN attenuates CIPN-induced mechanical allodynia in mice (Schwarz et al. 2024). Of note, locomotor effects were also observed at the dose used in the study, aligning with the effect of CBN observed here. In the present study, measures of mechanical allodynia were limited by hyperreflexia induced by CBN. Hyperreflexia is decreased muscle control that often precedes catalepsy induced by various cannabinoid agonists. The partial effects of CBN at CB_1_ are consistent with partial agonism previously reported in vitro (Booker et al. 2009). Regardless of the limitations induced by the observed hyperreflexia, it is unlikely that CBN will reduce mechanical allodynia at doses that do not also induce overt locomotor confounds.

The present study is the first to evaluate the in vivo effects of cannabicyclol (CBL), a degradative product of CBC formed via environmental irradiation (Crombie et al. 1968). In the present study, CBL induced hypothermia and hypolocomotion, each of which was blocked by adenosine A_2A_ receptor antagonism. These data indicate that CBL functions as a novel adenosine A_2A_ agonist in vivo. Similarly, CBL attenuation of CCI-induced cold allodynia occurred through a mechanism requiring A_2A_ activation. It is worth noting that contralateral paw lifting was also positively correlated with body temperature in the CBL-treated group (r^2^ = 0.56). Therefore, it cannot be ruled out that the observed anti-allodynia may be related to the hypothermic effects of CBL given that these effects were observed at doses that also induced mild hypothermia. That is, we cannot exclude the possibility that the lower body temperature of the mice may have impaired perception of the cold stimulus. Yet, CBL also blocked hind paw inflammation and the increase in paw tissue cytokine levels in the LPS model. A_2A_ receptors have well-established roles in immunity, being expressed on many immune cell types such as neutrophils, eosinophils, monocytes, lymphocytes, epithelial and endothelial cells, and macrophages (Guerrero 2018). It is a strong possibility that the anti-inflammatory effects of CBL could be mediated via A_2A_ activation. Although considerable effort has been devoted to developing adenosine receptor agonists as treatments for a number of diseases, many ultimately fail in clinical trials due to the production of undesirable effects. CBL, on the other hand, may have fewer or milder side effects, but more work is needed to determine its safety and usefulness in human health conditions.

Although CBN reduced cold allodynia, it is noteworthy that none of the minor cannabinoids tested affected CCI-induced mechanical allodynia. Cannabichromene (CBC), which previously exhibited weak antinociception in the tail-flick assay (Maione et al. 2011; Zagzoog et al. 2020), was ineffective here. In contrast, CBC attenuated cisplatin-induced mechanical and cold allodynia, formalin-induced spontaneous pain behaviors, and thermal hyperalgesia in the tail-flick assay (Raup-Konsavage et al. 2024). These discrepancies may reflect mechanistic differences between chemotherapy-induced peripheral neuropathy and CCI. Chemotherapy-induced peripheral neuropathy is a chemically-induced condition characterized by mitochondrial dysfunction, oxidative stress, and peripheral sensory neuron toxicity, whereas CCI is surgically-induced and involves nerve injury and central nociceptive pathways (Jali et al. 2024). Additional differences in experimental design, including route of administration, timing of treatment, or pain stimulus modality, may also contribute to the divergent effects of CBC. For example, one study delivered microinjections of CBC into the ventrolateral periaqueductal grey and measured tail-flick latency in response to radiant heat (Maione et al. 2011), whereas another study measured tail withdrawal latency from 52 °C water 15 min after i.p. injection (Zagzoog et al. 2020). Thus, it is plausible that CBC-induced analgesia is dependent upon local administration, a shorter incubation period, or type of thermal stimuli. Furthermore, the lack of efficacy in the CCI model observed here may suggest that CBC is more effective at treating chemically-induced peripheral sensitization than physical injury accompanied by central neuroimmune processes.

In the marble burying test, either CBN or CBL reduced the number of marbles buried (Thomas et al. 2009) (i.e., a proxy measure of digging behavior) but also induced immobility at the same doses, confounding any clear conclusion about potential anxiolysis. That is, CBN- and CBL-treated mice may have dug less during the test due to reduced locomotor activity. These data are consistent with previously reported effect of Δ^9^-THC in depression of both marble burying and locomotor activity (Kinsey et al. 2011a, b). Similarly, none of the minor cannabinoids tested displayed anti-depressive-like effects in the tail suspension test, even at relatively high doses. CBG increased the time male Sprague-Dawley rats spent in the open arms of the elevated plus maze (Mendiguren et al. 2023), interpreted as an anxiolytic-like effect. The elevated plus maze was not used here, due to its susceptibility to confound by myriad environmental factors (Shoji and Miyakawa 2021). For instance, increasing endocannabinoid levels via FAAH deletion increases time in the open arms, but this effect is dependent on test room lighting (Naidu et al. 2007). Regardless, the lack of an antidepressant-like effect in the present study aligns with a previous report that neither CBN nor CBG increases time struggling in the forced swim test (El-Alfy et al. 2010), a model of learned helplessness.

In the present study, no drug x sex interactions were observed in our preliminary analyses, with two exceptions. Female mice treated with CBL buried fewer marbles [F(5,46) = 2.55; *p* = 0.0407], and male mice treated with CBN struggled more in the tail suspension test [F(5,35) = 2.73; *p* = 0.0347]. These data may be interpreted as relative sex-based differences in sensitivity to either compound’s hypolocomotive effects but do not indicate a clear anxiolytic-like or anti-depressant-like pattern. Given the lack of a clear pattern across tests and the small sample size (*n* = 5/sex), we have reservations about the biological relevance of these potentially spurious results. Future studies powered to detect sex differences are needed.

Click or tap here to enter text.Click or tap here to enter text.Click or tap here to enter text.Availability and use of minor cannabinoid isolates have grown exponentially in recent years, although there remains a paucity of data evaluating their therapeutic efficacy. The antiedematous and anti-inflammatory potential of cannabinol and cannabicyclol reported here are encouraging, but much additional study, particularly regarding safety, is needed before appropriate recommendations can be made for any clinical application in people. Furthermore, CBN may be limited by the induction of psychoactive effects at high doses. As with many plant-derived compounds, these chemicals activate multiple different receptor systems and may have unpredictable effects, especially at higher doses or after repeated administration. For example, although some of the effects of CBN were partially blocked by CB_1_, adenosine A_2A_, or TRPV1 receptor antagonism, none of these receptors appear to fully mediate the antinociceptive, hypothermic, or locomotor effects of CBN. Thus, the potential side effect and drug interaction profile of minor cannabinoids is yet to be established, and more work is needed to uncover the pharmacological activity and molecular targets of these minor constituents found in cannabis.

## Supplementary Information

Supplementary Material 1.

## Data Availability

The authors declare that all the data supporting the findings of this study are contained within the paper.
