# Supplementary material for: Anti-inflammatory and analgesic potential of minor cannabinoids in vivo
Source: J Cannabis Res. 2026 Feb 12;8:40. doi: 10.1186/s42238-025-00384-7 (PMC12998089; doi:10.1186/s42238-025-00384-7)
Supplement: Supplementary file 1 — Supplementary Material 1. [file 42238_2025_384_MOESM1_ESM.docx]

**SUPPLEMENTARY MATERIALS AND METHODS**

**Marble burying test**

The marble burying test was performed in a separate group of naïve male C57BL/6J mice (n=10 [5M/5F]) as described in the primary manuscript. Mice were pretreated (i.p.) 1 h before testing with the benzodiazepine diazepam (0.3, 1, or 3 mg/kg) or the selective serotonin reuptake inhibitor fluoxetine (10, 20, or 30 mg/kg) to act as positive controls for anxiolytic and antidepressant-like behavior.

**Tail Suspension Test**

Immediately following the marble burying test, the tail suspension test was performed as described in the manuscript.

**Data and statistical analyses**

Data from the marble burying and tail suspension tests were analyzed using one-way ANOVA, followed by Bonferroni *post hoc* comparisons, with drug treatment as the between-subjects independent variable. All data are represented as mean ± SEM, and results were considered significant if p<0.05.

The analyses in the tables below were performed as described in the primary manuscript. Dose–response curves were fitted using nonlinear regression (four-parameter logistic model) in GraphPad Prism (version 10), and ED₅₀ and corresponding 95% confidence intervals were derived from the fitted curves. Effect sizes for ANOVA were expressed as partial eta squared (ηp²), computed from the F statistic as ηp² = (*F* × *df*_effect_) / (*F* × *df*_effect_ + *df*_error_).

**Fig. S1. Effects of clinically relevant anxiolytics on anxiety-like behavior in the marble burying and tail-suspension tests.** Diazepam (DZ; 1 mg/kg) or fluoxetine (FLX; 10-20 mg/kg) reduced **(A, D)** marble burying without increasing **(B, E)** time immobile in the marble burying test. **(F)** FLX (≥20 mg/kg), but not **(C)** diazepam, also increased struggling in the tail suspension test, consistent with reduced anxiety-like behavior. Data are expressed as mean ± SEM (n=7-8 male mice). *-***p<0.05-0.0005 vs vehicle control

| **Table S1. Tetrad dose-response** | | | | | | |
| --- | --- | --- | --- | --- | --- | --- |
|  |  | *F* (DF) | *p* | ED_50_ (95% CI) | n [M/F] | ηp² |
| Catalepsy | CBN | 11.8 (6,102) | **<0.0001** | 120.7 (89.4-162.9) | 10 [5/5] | .41 |
|  | CBC | 0.1 (5,80) | 0.99 |  | 9 [5/4] | .01 |
|  | CBL | 1.9 (5,85) | 0.10 |  | 10 [5/5] | .10 |
| Antinociception | CBN | 8.8 (5,85) | **<0.0001** | 206.6 (142.7-299) | 10 [5/5] | .34 |
|  | CBC | 0.6 (4,64) | 0.69 |  | 9 [5/4] | .04 |
|  | CBL | 0.2 (4,68) | 0.92 |  | 10 [5/5] | .01 |
| Hypothermia | CBN | 32.3 (6,102) | **<0.0001** | 101.3 (88-116.6) | 10 [5/5] | .66 |
|  | CBC | 2.6 (5,80) | **0.0364** | >500 | 9 [5/4] | .14 |
|  | CBL | 7.5 (5,85) | **<0.0001** | 78.1 (55.7-109.5) | 10 [5/5] | .31 |
| Immobility | CBN | 10.7 (5,44) | **<0.0001** | 59.6 (40.3-88.2) | 8 [4/4] | .55 |
|  | CBC | 7.8 (5,44) | **<0.0001** | >500 | 8 [4/4] | .47 |
|  | CBL | 3.8 (5,44) | **0.0062** | 210.6 (136.5-324.8) | 8 [4/4] | .30 |
| Summary of main effects corresponding to Fig. 1.A-D. ED_50_ values are listed in mg/kg. A *p*-value of less than 0.05 is marked in bold. CBN, cannabinol; CBC, cannabichromene; CBL, cannabicyclol. | | | | | | |

| **Table S2. CBN tetrad time course** | | | | | |
| --- | --- | --- | --- | --- | --- |
|  |  | *F* (DF) | *p* | n [M/F] | ηp² |
| Catalepsy | CBN | 13.8 (6,108) | **<0.0001** | 10 [5/5] | .43 |
| Antinociception | CBN | 4.2 (5,87) | **0.0018** | 9-10 [4-5/5] | .19 |
| Hypothermia | CBN | 20.8 (6,108) | **<0.0001** | 10 [5/5] | .54 |
|  | CBL | 9.7 (4,72) | **<0.0001** | 10 [5/5] | .35 |
| Summary of main effects corresponding to Fig. 1.E-H. A *p*-value of less than 0.05 is marked in bold. CBN, cannabinol; CBL, cannabicyclol. | | | | | |
|  |  |  |  |  |  |

| **Table S3. Tetrad antagonist challenge** | | | | | |
| --- | --- | --- | --- | --- | --- |
|  |  | *F* (DF) | *p* | n [M/F] | ηp² |
| Catalepsy | rim | 10.1 (6,63) | **<0.0001** | 10 [5/5] | .49 |
|  | istr | 3.5 (6,61) | **0.0051** | 9-10 [4-5/5] | .26 |
|  | CPZ | 5.4 (2,27) | **0.0104** | 10 [5/5] | .29 |
| Antinociception | rim | 18.2 (6,63) | **<0.0001** | 10 [5/5] | .63 |
|  | istr | 4.4 (6,61) | **0.001** | 9-10 [4-5/5] | .30 |
|  | CPZ | 2.9 (2,27) | 0.07 | 10 [5/5] | .18 |
| Hypothermia | rim | 8.4 (6,63) | **<0.0001** | 10 [5/5] | .44 |
|  | istr | 26.5 (6,61) | **<0.0001** | 9-10 [4-5/5] | .72 |
|  | CPZ | 51.0 (2,27) | **<0.0001** | 10 [5/5] | .79 |
| Immobility | rim | 9.1 (6,63) | **<0.0001** | 10 [5/5] | .46 |
|  | istr | 48.7 (6,61) | **<0.0001** | 9-10 [4-5/4-5] | .83 |
|  | CPZ | 16.3 (2,27) | **<0.0001** | 10 [5/5] | .55 |
| Summary of main effects corresponding to Fig. 2. A *p*-value of less than 0.05 is marked in bold. rim, rimonabant (CB_1_-selective antagonist); istr, istradefylline (A_2A_-selective antagonist); CPZ, capsazepine (TRPV1-selective antagonist). | | | | | |

| **Table S4. LPS-induced hindpaw inflammation** | | | | | |
| --- | --- | --- | --- | --- | --- |
|  |  | *F* (DF) | *p* | n [M/F] | ηp² |
| Paw thickness |  | 40.0 (6,55) | **<0.0001** | 8-9 [4-5/3-5] | .81 |
| Thermal Preference | | 5.7 (6,55) | **0.0001** | 8-9 [4-5/3-5] | .38 |
| Distance (TGR) |  | 36.7 (6,55) | **<0.0001** | 8-9 [4-5/3-5] | .80 |
| Cytokines | IL-6 | 4.5 (6,53) | **0.0009** | 8-9 [4-5/3-5] | .34 |
|  | IL-1β | 9.2 (6,53) | **<0.0001** | 8-9 [4-5/3-5] | .51 |
|  | TNF-α | 4.4 (6,51) | **0.0011** | 8-9 [4-5/3-5] | .34 |
| Summary of main effects corresponding to Fig. 4. A *p*-value of less than 0.05 is marked in bold. TGR, thermal gradient ring; IL-6, interleukin-6; IL-1β, interleukin-1 beta; TNF-α, tumor necrosis factor alpha. | | | | | |

| **Table S5. CCI-induced neuropathic pain** | | | | | | |
| --- | --- | --- | --- | --- | --- | --- |
|  |  | *F* (DF) | *p* | ED_50_ (95% CI) | n [M/F] | ηp² |
| von Frey | CBN | 0.9 (4,45) | 0.45 |  | 10 [5/5] | .07 |
|  | CBC | 2.2 (5,54) | 0.07 |  | 10 [5/5] | .17 |
|  | CBL | 1.8 (5,54) | 0.14 |  | 10 [5/5] | .14 |
| Acetone | CBN | 13.1 (4,45) | **<0.0001** | 82.0 (68.4-98.2) | 10 [5/5] | .54 |
|  | CBC | 4.3 (5,54) | **0.0023** | >500 | 10 [5/5] | .28 |
|  | CBL | 6 (5,54) | **0.0002** | 21.3 (10.1-45.0) | 10 [5/5] | .36 |
|  | rim/SR2+CBN | 6.1 (3,36) | **0.0018** |  | 10 [5/5] | .34 |
|  | istr+CBL | 6.8 (2,27) | **0.0042** |  | 10 [5/5] | .33 |
| Body Temp | istr+CBL | 11.9 (2,27) | **0.0002** |  | 10 [5/5] | .47 |
| Hyperreflexia | CBN | 8.5 (3,36) | **0.0002** |  | 10 [5/5] | .41 |
| Summary of main effects corresponding to Fig. 4. ED_50_ values are listed in mg/kg. A *p*-value of less than 0.05 is marked in bold. CBN, cannabinol; CBC, cannabichromene; CBL, cannabicyclol; rim, rimonabant (CB_1_-selective antagonist); SR2, SR144528 (CB_2_-selective antagonist); istr, istradefylline (A_2A_-selective antagonist). | | | | | | |

| **Table S6. Anxiety- and depressive-like behaviors** | | | | | |
| --- | --- | --- | --- | --- | --- |
| Light/dark box |  | *F* (DF) | *p* | n [M/F] | ηp² |
| Time in light | CBN | 1.6 (5,42) | 0.19 | 8 [4/4] | .16 |
|  | CBL | 0.4 (5,54) | 0.84 | 10 [5/5] | .04 |
|  | CBG | 1.1 (5,54) | 0.38 | 10 [5/5] | .09 |
| Latency | CBN | 1.2 (5,42) | 0.31 | 8 [4/4] | .13 |
|  | CBL | 1.8 (5,54) | 0.11 | 10 [5/5] | .14 |
|  | CBG | 0.4 (5,54) | 0.83 | 10 [5/5] | .04 |
| Distance | CBN | 4.3 (5,43) | **0.0032** | 8 [4/4] | .33 |
|  | CBL | 6.6 (5,54) | **<0.0001** | 10 [5/5] | .38 |
|  | CBG | 1.5 (5,54) | 0.20 | 10 [5/5] | .12 |
| Marble burying |  |  |  |  |  |
| Marbles buried | CBN | 31.9 (5,54) | **<0.0001** | 10 [5/5] | .75 |
|  | CBL | 14.5 (5,52) | **<0.0001** | 9-11 [4-6/4-6] | .58 |
|  | CBG | 2.3 (5,54) | 0.06 | 10 [5/5] | .18 |
| Time immobile | CBN | 81.7 (5,54) | **<0.0001** | 10 [5/5] | .88 |
|  | CBL | 15.6 (5,52) | **<0.0001** | 9-11 [4-6/4-6] | .60 |
|  | CBG | 4.6 (5,54) | **0.0014** | 10 [5/5] | .30 |
| Tail suspension | CBN | 7.3 (5,42) | **<0.0001** | 8 [4/4] | .46 |
|  | CBL | 2.2 (5,54) | 0.06 | 10 [5/5] | .17 |
|  | CBG | 0.9 (5,54) | 0.50 | 10 [5/5] | .08 |
| Summary of main effects corresponding to Fig. 5. A *p*-value of less than 0.05 is marked in bold. CBN, cannabinol; CBL, cannabicyclol; CBG, cannabigerol. | | | | | |
